# Microbial Growth Inhibition Effect, Polyphenolic Profile, and Antioxidative Capacity of Plant Powders in Minced Pork and Beef

**DOI:** 10.3390/foods13193117

**Published:** 2024-09-29

**Authors:** Kadrin Meremäe, Linda Rusalepp, Alar Sünter, Piret Raudsepp, Dea Anton, Mihkel Mäesaar, Terje Elias, Tõnu Püssa, Mati Roasto

**Affiliations:** Chair of Veterinary Biomedicine and Food Hygiene, Institute of Veterinary Medicine and Animal Sciences, Estonian University of Life Sciences, Fr. R. Kreutzwaldi 56/3, 51006 Tartu, Estonia

**Keywords:** antimicrobial activity, antioxidative activity, microbial growth dynamics, minced pork, minced beef, pomaces, plant powders, polyphenolic profile

## Abstract

Consumer interest in healthier meat products has grown in recent years. Therefore, the use of plant powders as natural preservatives in the composition of pork and beef products could be an alternative to traditional meat products. This study aimed to assess the effect of different powders, such as blackcurrant, chokeberry, rowan berries, apple, tomato, garlic, and rhubarb, on the microbial growth dynamics in minced pork and beef during refrigerated storage. The total counts of aerobic microorganisms, *Pseudomonas* spp., yeasts, and molds were examined according to ISO methods. The polyphenolic profiles of plant powders and supplemented minced pork and beef samples were determined by HPLC-MS. The antioxidative capacity of the plant powders was analyzed using a spectrophotometric method. The findings of the study revealed that supplemented minced pork and beef samples had similar polyphenolic profiles and microbial growth dynamics. The highest antioxidative capacity was observed for anthocyanin-rich berry powders. In both minced pork and beef, rhubarb powder was the most effective plant material for inhibiting microbial growth, followed by blackcurrant pomace powder. In conclusion, all of the plant powders used in the present study can be used for the valorization of minced meat products, providing both antimicrobial and antioxidant effects.

## 1. Introduction

A balanced diet, which includes both plant- and animal-origin products, is important in human nutrition [1]. Both pork and beef contain high levels of protein, fats, minerals, and vitamins. However, since meat is a favorable growth environment for microorganisms, it is considered an easily perishable food [2]. In addition, minced meat products are more susceptible to microbial contamination, because mincing increases the surface area of the meat, which can be exposed to microbes [3]. Oxidative processes and microbial growth during storage causes meat spoilage and shortens the shelf life of raw meat and meat products [4]. Total counts of microorganisms indicate the level of meat production hygiene and the microbiological quality of the products. Therefore, microbial counts can be used in the determination of meat products’ shelf life [5]. Different genera and species of microorganisms, particularly *Pseudomonas* spp. can grow in meat; therefore, its counts are often used as indicator of microbial spoilage in minced meat and raw meat products [6].

The growing consumer interest in healthier foods has resulted in research in using plant-based preservatives for the valorization of the animal-origin products [7]. Plants are natural sources of polyphenols and other phytochemicals with antimicrobial and antioxidant properties; therefore, they can act as natural preservatives in foods [8,9]. Various fruits and berries are good sources of polyphenols [10,11,12]. Plant polyphenols with antioxidant and antimicrobial properties can reduce microbial growth and extend the shelf life of meat and meat products [4,13,14]. Also, pomaces from juice production are often a valuable source of polyphenols [15]. In a recent study [16], the use of fruit and berry pomaces in the composition of marinades effectively inhibited the growth of *L. monocytogenes* in raw fish. Therefore, the plant pomaces obtained from fruit and vegetable processing due to their rich composition of bioactive compounds have good potential for use in food production as natural food compounds with antioxidant and antimicrobial activity [17].

Many studies have evaluated the antioxidant and antimicrobial activity of plant extracts in vitro [11,18,19]. However, there are qualitative and quantitative variations in their effectiveness, depending on the content of bioactive substances in plant materials and the technologies of how the plant materials are prepared and used in food products [20]. Therefore, the antimicrobial effects of plant powders in food products must also be scientifically proven considering a feasible food shelf life.

There are only a few published studies on the enrichment of meat products with plant powders. These studies have proved both antimicrobial and antioxidative effect of selected plant powders in animal-origin food products [8,14,21]. Also, sensory evaluations gave promising results in Anton et al. [14] and Kerner et al.’s [21] studies. The findings are important for the food industry, where the addition of natural supplements into meat products will meet consumers’ increasing demand for more natural and healthy products.

The aim of this study was to evaluate the microbial growth inhibition effect, polyphenolic profile, and antioxidative capacity of selected plant powders in minced pork and beef.

## 2. Materials and Methods

### 2.1. Minced Meat

Fresh minced pork with a fat content of 28% and minced beef with a fat content of 18% were used in this study. Minced meats from a large-scale Estonian meat factory were purchased as fresh as possible and transported to the laboratory in cooled transport boxes at a temperature of 2 °C. The experimental samples were prepared instantly, and analyses started after overnight storage in the refrigerator.

### 2.2. Plant Material and Preparation of Powders

The pomace powders of berries of blackcurrant (BC, *Ribes nigrum* L.), chokeberry (CB, *Aronia melanocarpa* (Michx.) Elliott), rowan (RB, *Sorbus* sp.), tomato (TO, *Solanum lycopersicum* L.), and petioles of rhubarb (RHB, *Rheum rhaponticum* L.) were used. In addition, the powders of bulbs of garlic (GAR, *Allium sativum* L.) (Santa Maria AS, Saue, Estonia) and apples were used (*Malus domestica* Borkh.). In the case of apples, two variants of powders were used: powder prepared from whole apples (APLS) and powder of apples from which the seeds were removed (APL). Pomaces and powders were prepared at the Polli Horticultural Research Center and Food Hygiene Department of the Estonian University of Life Sciences. Plant material was dried at 50 °C using a Binder FED 115 drying chamber (Binder GmbH, Tuttlingen, Germany) or an Alpfrigo CFD 1400 SS condensation fruit dryer (Alpfrigo d.o.o., Logatec, Slovenia). The dried material was milled into a fine powder using a Motoria KM855 grinder (Komanda Technics Europe, England, London) or a Retsch GM 300 grinding mill (Retsch GmbH, Haan, Germany), and sieved to obtain a fraction of ≤1 mm. The amount of the plant powder was 2% (*w*/*w*) of the minced meat, based on the sensory evaluation results, which indicated that the minced meat samples supplemented with the selected powders were acceptable.

### 2.3. Determination of Antioxidative Capacity of Plant Powders

The antioxidative (AO) capacity of plant powders was determined as described by Meremäe et al. [18]. In brief, analyses of free radical (DPPH) scavenging capacity of diluted plant extracts in 60% EtOH were performed using the DPPH free radical scavenging method on a plate reader, namely the Infinite 200 Pro M Plex Mono Cuvette instrument (Tecan Austria Gmbh, Grödig, Austria). Measurements were performed at 515 nm on duplicate plates, using 50 µL of sample and 150 µL of DPPH (1 mM) solution per well. Furthermore, 60% EtOH was used as a blank sample. For the quantification of antioxidative capacity, a Trolox calibration curve was used, measured at the same conditions as the samples. The results are expressed in Trolox equivalents per gram of dry weight of the plant powders.

### 2.4. Determination of Total Phenolic Content

The total phenolic content (TPC) of plant powder extracts in 60% aqueous ethanol was determined spectrophotometrically using the Folin–Ciocalteu method described by Waterhouse [22]. Briefly, 20 µL of plant extract was mixed with 1.58 mL deionized water and 100 µL Folin–Ciocalteu reagent in cuvettes. After 8 min, 300 µL sodium carbonate solution was added, mixed, and incubated at room temperature for 2 h. The absorbance was measured at 765 nm using an Infinite 200 Pro M Plex Mono Cuvette instrument (Tecan Austria Gmbh). Gallic acid was used as the standard, and the results were expressed as gallic acid equivalent (GAE) milligrams per gram of dry weight.

### 2.5. Preparation of Minced Meats with Plant Powders

Two percent of plant powders were added to the minced meats, and a Clatronic HM 2935 (Clatronic International GmbH, Kempen, Germany) mixer was used to thoroughly mix the minced meat with the plant powders. Mixing of all samples was carried out for three minutes at the first speed level. Pure minced meat was used as a control. At least 150 g of sample material was stored per each sample per each time point. Samples were packed in sterile screw-on lid cups and stored in the refrigerator at 5 ± 1 °C for a maximum of 8 days (except for RHB, which was kept for 14 days). All analyses of the samples were performed in duplicate on days 1, 4, 6, and 8 and on days 11 and 14 for RHB, which especially strongly inhibited microbial growth.

### 2.6. Enumeration of Microorganisms

For the enumeration of total microbial counts, *Pseudomonas* spp., and yeasts and molds, the laboratory protocols were used according to EVS-EN ISO standards 4833-2:2013/A1:2022 [23], 13720:2010 [24], and EVS-ISO 21527-1:2009 [25]. A detailed description of the microbiological analyses is given in the study of Koskar et al. [8]. Colonies were counted after incubation at specified temperatures, and the results were reported as log colony-forming units per gram (cfu/g).

### 2.7. Estimation of pH and Water Activity

For pH determination, the sample was diluted with distilled water at a ratio of 1:10, followed by homogenization and filtration. The pH of the samples was determined using a digital pH meter (HandyLab680 (SI Analytics GmbH, Mainz, Germany). Water activity (a_w_) was determined at 25 °C using an Aqualab Decagon 4TE (Decagon Devices Inc., Pullman, WA, USA) following the manufacturer’s instructions.

### 2.8. Chromatographic Analyses

Plant powders (0.5 g) were weighed into tubes and 5 mL 60% aqueous ethanol was added, mixed, shaken at 60 rpm for 60 min in a Multi RS-60 Multirotator (BioSan, Riga, Latvia), and sonicated for 30 min. The extracts were centrifuged at 3200× *g* for 10 min using an Eppendorf 5810R (Eppendorf AG, Hamburg, Germany) centrifuge, and the supernatant was used for chromatographic analysis.

Minced meat samples supplemented with plant powders were extracted after 24 h and 8 days of refrigeration. Then 4 mL of methanol was added to 2 g of sample and shaken for 30 min in a Multi RS-60 Multirotator (BioSan) and centrifuged at 3200× *g* for 10 min using an Eppendorf 5810R (Eppendorf AG) centrifuge. The supernatant was extracted twice with 2 mL of hexane to remove the fat. The hexane phases were discarded, and the methanol phase was passed through a C18 SPE column (Agilent Technologies, Waldbronn, Germany) for further chromatographic analyses.

The samples were analyzed using a 1290 Infinity system (Agilent Technologies), coupled to an Agilent 6450 Q-TOF mass spectrometer equipped with a JetStream ESI source, and to an Agilent 1290 Infinity Diode Array Detector, as previously described by Meremäe et al. [18]. Data acquisition and initial data processing were carried out using MassHunter software (MH Data Acquisition v. B.09.00; MH Qualitative Analysis v. 10.0, Agilent Technologies). Compounds were identified by comparison of the *m*/*z* values, retention times, UV spectra, and MS/MS fragmentation patterns with standards or by comparing data from the literature or the METLIN database (v. B.02.00, Agilent Technologies).

### 2.9. Statistical Analyses

Microsoft Excel 365 (Microsoft Corporation; Redmond, WA, USA) was used for data collection and preliminary statistical analyses, including the calculation of means and standard deviation (SD). Further statistical analysis was performed using JASP (Version 0.18.3) [26]. Dunn’s post hoc pairwise comparisons using Bonferroni adjustments after Kruskal–Wallis’s test were performed to determine significant differences (*p* < 0.05) in the total counts of microorganisms between controls and minced pork and beef samples containing different plant powders. Correlations (r) between the variables, such as the total microbial counts, pH, a_w_, TPC, and AO of tested samples, were calculated using the Pearson correlation. Correlations were performed between the AO and TPC of plant materials in two ways: when all plant materials were included in the correlations, and when samples with chokeberry and tomato additives were excluded. The correlation was significant at the *p* < 0.05 level.

## 3. Results

### 3.1. Total Microbial Counts in Minced Pork and Minced Beef

**Figure 1:**
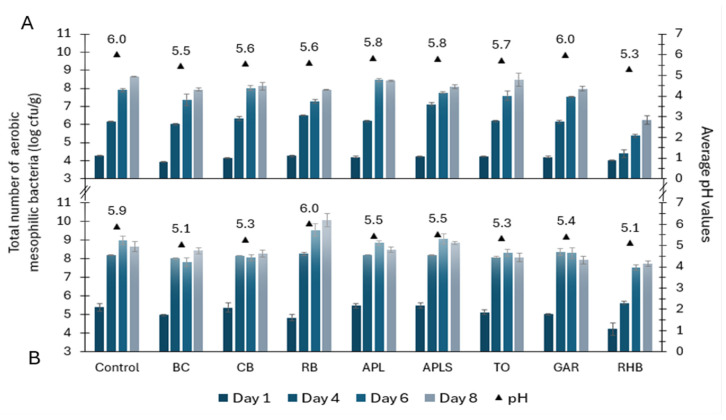
Microbial growth dynamics (counts in log cfu/g ± SD) and average pH values in minced pork (**A**) and beef (**B**) in controls and samples supplemented with plant powders, such as blackcurrant (BC), chokeberry (CB), rowan berries (RB), apple, seeds removed (APL), apple with seeds (APLS), tomato (TO), garlic (GAR), and rhubarb (RHB).

The total counts of aerobic mesophilic microorganisms in minced pork and beef samples were 3.95–4.29 and 4.23–5.50 log cfu/g on day 1 (Figure 1). Of the tested pork samples, both BC (*p* < 0.001) and RHB (*p* < 0.01) samples had significantly lower counts compared to the control samples containing 3.95 ± 0.04 and 4.04 ± 0.04 log cfu/g, respectively. The rest of the pork and beef samples supplemented with plant materials were not significantly different from the control samples on day 1. On the fourth and sixth day, there was a further increase in the total counts of microorganisms in both types of minced meat samples. However, the microbial count in rhubarb samples remained lower than in others, especially in the minced pork.

On the eighth day, the total counts of microorganisms in the minced pork samples ranged from 7.91 to 8.65 log cfu/g except for the RHB sample (6.24 ± 0.14 log cfu/g), which contained significantly (*p* < 0.001) less bacteria than the control sample. Mean bacterial counts on day 8 in minced beef samples were between 7.74 and 10.06 log cfu/g. Compared to the control sample, the total microbial counts were also slightly lower in RHB minced beef samples but were not significantly different from the control. On day 14, analyses were performed only for RHB samples. The total microbial counts in RHB samples were 8.34 ± 0.10 log cfu/g for minced pork samples and 9.10 ± 0.03 log cfu/g for minced beef samples, which were similar to the results of the other samples on day 8.

### 3.2. Counts of Pseudomonas spp. in Minced Pork and Minced Beef

**Figure 2:**
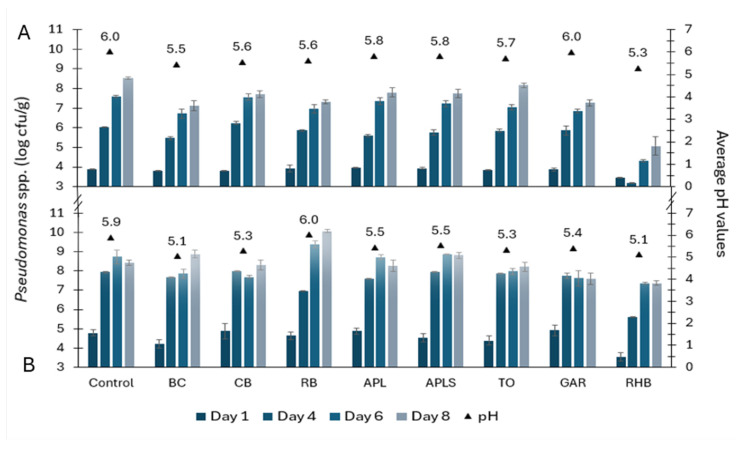
*Pseudomonas* spp. growth dynamics (counts in log cfu/g ± SD) and average pH values in minced pork (**A**) and beef (**B**) in controls and samples supplemented with plant powders, such as blackcurrant (BC), chokeberry (CB), rowan berries (RB), apple, seeds removed (APL), apple with seeds (APLS), tomato (TO), garlic (GAR), and rhubarb (RHB).

The counts of *Pseudomonas* spp. in minced pork and beef samples were 3.45–3.94 and 3.54–4.92 log cfu/g on day 1, respectively (Figure 2). In the following days, there was a continuous increase in the counts of *Pseudomonas* spp. in both minced meat samples. However, RHB samples contained significantly less *Pseudomonas* bacteria compared to the control samples (*p* < 0.05 on day 4 in both minced pork and beef samples and *p* < 0.01 on day 6 only in minced pork samples). On day 6, in minced pork with BC, the counts of *Pseudomonas* spp. were significantly lower compared to the control (*p* < 0.05).

On day 8 of the durability study, the counts of tested bacteria ranged from 5.08 to 8.53 log cfu/g in pork samples and 7.37 to 10.07 log cfu/g in beef samples, respectively. The most significant difference in the counts of *Pseudomonas* spp. of the minced pork was between the control and the RHB sample (*p* < 0.001), followed by the BC sample, but no similar inhibitory effect was found for minced beef samples. However, the counts of *Pseudomonas* spp. were slightly lower in RHB-supplemented minced beef samples compared to the others. On day 14, RHB pork and beef samples contained 6.12 ± 0.12 log cfu/g and 7.30 ± 0.35 log cfu/g of *Pseudomonas* spp., respectively.

### 3.3. Yeasts and Molds Counts in Minced Pork and Minced Beef

**Figure 3:**
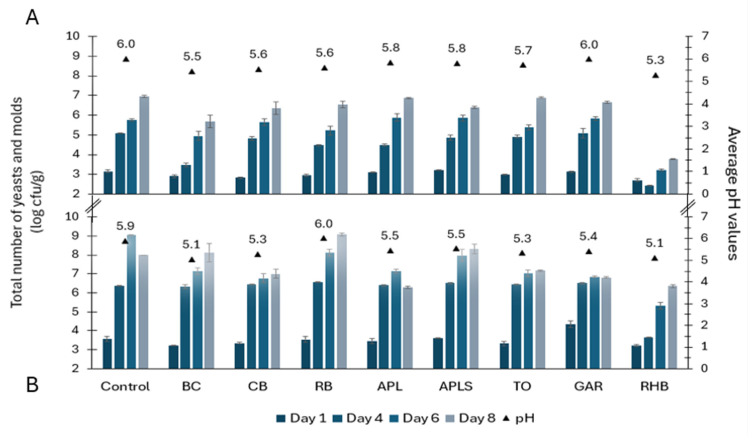
Yeasts and molds growth dynamics (counts in log cfu/g ± SD) and average pH values in minced pork (**A**) and beef (**B**) in controls and samples supplemented with plant powders. Abbreviations: blackcurrant (BC), chokeberry (CB), rowan berries (RB), apple, seeds removed (APL), apple with seeds (APLS), tomato (TO), garlic (GAR), and rhubarb (RHB).

On day 1, the total counts of yeasts and molds ranged from 2.71 to 3.16 log cfu/g in minced pork samples and from 3.23 to 4.33 log cfu/g in minced beef samples (Figure 3). In the following days, the counts of tested microorganisms increased steadily in all samples. On day 8, the count of yeasts and molds in minced pork was 6.54 ± 0.45 log cfu/g on average. Among minced beef samples, there was greater variability in microbial counts, with an average of 6.81 to 9.07 log cfu/g. The total counts of yeasts and molds remained lower in both types of RHB-supplemented minced meat and BC-supplemented minced pork samples compared to the controls and other samples throughout the study period. On day 14, RHB pork and beef samples contained 4.92 ± 0.03 log cfu/g and 7.0 ± 0.15 log cfu/g of yeasts and molds counts, respectively.

### 3.4. Ranking of Meat Samples Based on Microbial Counts

**Figure 4:**
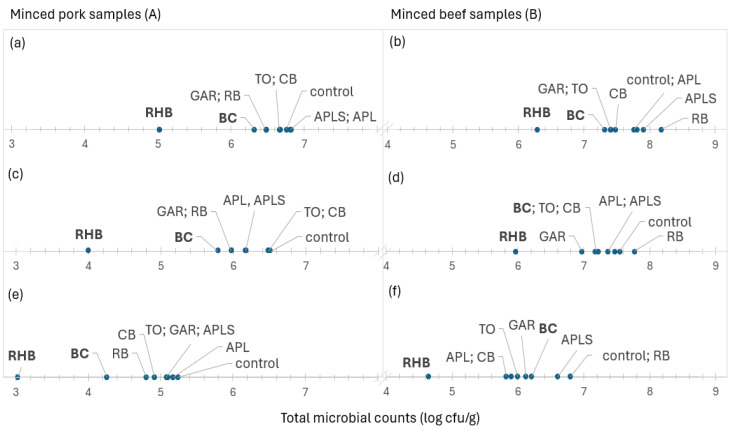
The average total microbial counts (**a**,**b**), *Pseudomonas* spp. (**c**,**d**), and yeasts and molds (**e**,**f**) arranged in ascending order from left to right in minced pork (**A**) and beef (**B**) samples in control samples and samples supplemented with plant powders. Bold font highlights the two most effective supplements based on their effect on total microbial counts. Abbreviations: blackcurrant (BC), chokeberry (CB), rowan berries (RB), apple, seeds removed (APL), apple with seeds (APLS), tomato (TO), garlic (GAR), and rhubarb (RHB).

Figure 4 shows the ranking of minced meat samples supplemented with plant powders based on the mean total microbial counts from lowest to highest. The average total microbial counts and *Pseudomonas* spp. in minced pork (5.01 and 4.01 log cfu/g, respectively) and beef (6.28 and 5.97 log cfu/g, respectively) samples were lowest in the RHB samples, followed by the BC and GAR samples. The average total counts of yeasts and molds in minced pork and beef samples were also lower in the RHB (3.02 log cfu/g) and BC (4.30 log cfu/g) samples for minced pork, and in the RHB (4.64 log cfu/g) sample for minced beef, respectively. RHB powder in minced meat reduced the average total microbial counts by 10-fold, and the counts of *Pseudomonas* spp., yeasts, and molds by 100-fold compared to the control sample. Also, samples containing powders, such as BC, GAR, TO, and CB, showed lower total microbial counts than the controls. However, the total microbial counts were highest in all minced beef samples supplemented with RB. The highest microbial counts in minced pork samples varied but were higher in the samples supplemented mainly with APL and APLS. Correlation analysis only showed that there was a strong positive correlation between the total counts of yeasts and molds and *Pseudomonas* spp. in minced pork (r = 0.95; *p* < 0.001) and beef (r = 0.93; *p* < 0.001).

### 3.5. pH and Water Activity in Minced Pork and Minced Beef

The lowest average pH values in minced pork and beef samples were in the RHB (5.2 ± 0.14), BC (5.3 ± 0.28), CB (5.5 ± 0.21), and TO (5.5 ± 0.28) samples (Figure 1, Figure 2 and Figure 3). In the remaining samples, such as APL, APLS, and GAR, the average pH was 5.7 ± 0.21. The highest mean pH values were detected in the RB (5.8 ± 0.28) and control samples (6.0 ± 0.07). Correlation analysis showed a strong positive correlation between the total counts of yeasts and molds and the pH of minced pork (r = 0.86, *p* < 0.01) and minced beef (r = 0.73, *p* < 0.05). Additionally, there was a positive correlation between the counts of *Pseudomonas* spp. and the pH of minced pork and beef (r = 0.71; *p* < 0.05). The average water activity values were the highest in the control and RB (0.988 ± 0.002), followed by BC and GAR (0.987 ± 0.001), APL and CB (0.986 ± 0.0004), TO and RHB (0.986 ± 0.002), and APLS (0.985 ± 0.0012).

### 3.6. Polyphenolic Profiles of the Plant Powders and Supplemented Minced Meat Samples

In addition to organic acids, 27 different polyphenolic compounds belonging to the classes of anthocyanins, dihydrochalcones, flavanols, flavonols, and hydroxycinnamic acids were tentatively identified in the plant powders (Table 1). The most polyphenolic compounds were found in BC, CB, and RB powders with 20 compounds, followed by RHB powder with 16 different compounds. Phloridzin, quercetin, quercetin rhamnoside, a number of quercetin hexosides, quercetin rhamnosyl hexoside, and protocatechuic acid were found in all powders except GAR, where only caffeic acid glycosides were identified. Delphinidin glucoside, delphinidin rutinoside, and (epi)gallocatechins were identified only in BC powder. Two organic acids—citric and quinic acid—were also detected in all powders (except for quinic acid in GAR powder). Citramalic acid was found only in apple powders. Similar to the plant powders, the same organic acids, and polyphenolic compound classes were also detected in minced pork and beef samples. However, some compounds found in powders, e.g., procyanidin C-type and kaempferol rutinoside, were not detected in minced meat samples. Quercetin was detected in RHB powder and RHB-supplemented minced pork on day 1, but not on day 8.

**Figure 5:**
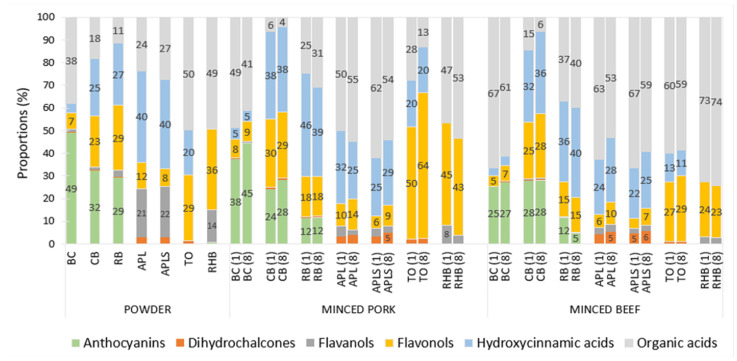
Relative content of classes of polyphenols and organic acids in plant powders and in supplemented minced pork and beef on days (1) and (8). Abbreviations: blackcurrant (BC), chokeberry (CB), rowan berries (RB), apple, seeds removed (APL), apple with seeds (APLS), tomato (TO), and rhubarb (RHB). Compounds with a relative content of less than 5% are not represented by a value in the figure.

The highest relative content of anthocyanins was found in the berry powders (29–49%), the highest relative hydroxycinnamic acids content in apple powders (40%), and the highest relative flavonols content in TO (29%) and RHB (36%) powders (Figure 5). The relative content of organic acids varied among plant powders, being highest (38–50%) in TO and RHB, followed by BC powders (Figure 5).

In both minced pork and beef samples, relatively the most anthocyanins were found in BC, the most hydroxycinnamic acids were found in CB, RB, APL, and APLS, and the most flavonols were found in TO and RHB. The highest relative content of organic acids (100%) was detected in the minced meat samples with GAR, followed by minced pork samples supplemented with apples and RHB (50–62%), and minced beef samples with RHB, BC, apples and TO (53–62%).

### 3.7. Antioxidative Capacity of Plant Powders

Table 2 shows the results of the free radical DPPH (2,2-diphenyl-1-picrylhydrazyl) scavenging assay and total phenolic content (TPC) of the different plant powders. Berry powders had the highest AO activity (119–130 mM TE/DWg), followed by apple powders (104–105 mM TE/DWg) and RHB powder (88 ± 9.2 mM TE/DWg). TPC was highest in CB powder (55.0 ± 1.29 mg GAE/DWg), followed by BC (24.6 ± 0.07 mg GAE/DWg) and RB powders (19.9 ± 0.06 mg GAE/DWg).

## 4. Discussion

The microbiological quality and shelf life of minced meat products directly depend on the counts of microorganisms, particularly of specific spoilage microorganisms, such as *Pseudomonas* spp., for meat products. The European Commission Regulation No. 2073/2005 [27] has set the limits for the aerobic colony count in minced meat. According to the process hygiene criteria of this regulation, the total microbial counts should not exceed 6.70 log cfu/g for two subsamples and 5.70 log cfu/g for three subsamples. In this study, at the beginning of the experiment, the average total microbial counts in the control samples of minced beef and pork were lower (5.3 and 4.3 log cfu/g, respectively) than the aforementioned limits, and this is characteristic for minced meat of good microbiological quality.

In this study, the analyses of minced pork and beef supplemented with different plant powders showed in most samples an average of 8−9 log cfu/g of aerobic mesophilic microorganisms at the last day of storage. A similar trend of increasing counts was detected for *Pseudomonas* spp. and yeasts and molds, but the total counts of yeasts and molds were on average ten times lower. Study results revealed that microbial growth dynamics were generally similar in minced pork and beef samples during the determined study period with a few exceptions. RHB pomace had the best microbial growth inhibition effect against aerobic mesophilic microorganisms, *Pseudomonas* spp., and yeasts and molds in both minced pork and beef samples. The second most effective pomace was BC, especially in minced pork samples. It can be explained by the fact that several bioactive compounds, including anthocyanins, flavanols, and flavonols were detected in RHB powder. All these polyphenols have antimicrobial properties [28].

In this study, the strong positive correlation between the counts of *Pseudomonas* spp., yeasts and molds and the pH of the minced meat samples also suggests a role for pH in influencing the count of microorganisms in the minced meat samples. The microbial growth inhibition effect of RHB powder in this study can also be explained by the lower pH and the presence of organic acids found in the minced pork and beef samples. According to Carpenter and Broadbent [29] organic acids inhibit bacterial cell growth by lowering cytoplasmic pH and causing osmotic stress, and because of the toxicity of organic acid anions. The indication of the presence of organic acids in the samples is pH, which was the lowest in RHB-containing meat samples, with average pH values of 5.1 in minced beef and 5.3 in minced pork (Figure 1, Figure 2 and Figure 3). Furthermore, RHB-supplemented samples showed extended stability on days 11 and 14, whereas the microbiological quality indicators for the other samples showed clear spoilage by day 8. According to these findings, the addition of RHB prolonged the microbiological shelf life of minced meat products. However, the AO capacity of RHB was low compared with the respective capacity of BC powder (88 and 130 mM TE/DWg, respectively). This finding is in agreement with the studies by Raudsepp et al. [11] and Püssa et al. [30] suggesting that rhubarb is high in polyphenols from anthraquinones and stilbenes, which are known for their strong antimicrobial properties but have weak antioxidant activity. Therefore, the properties and effectiveness of plant material depends on the content and diversity of the bioactive substances it contains [20]. 

In the present study, the richest composition of polyphenols was established in the berries of BC, CB, and RB, of which BC also showed inhibition of microbial growth in both minced pork and beef samples. BC has also shown strong antibacterial properties in a study by Trajković et al. [31], where lyophilized fruit juice and waste extract had moderate antimicrobial effects against the Gram-positive bacteria *B. cereus*, *E. faecalis*, and *S. aureus*. According to Kranz et al. [32], blackcurrant juice was the most effective whole fruit juice in inhibiting both Gram-positive and Gram-negative pathogenic bacteria of all the juices that were tested. Furthermore, Cho et al. [33] found that blackcurrant juice was a good natural additive for improving meat quality by inhibiting bacterial growth and biogenic amines formation, also hindering the increase in pH at refrigerated temperatures. In the present study, the antimicrobial activity of BC pomace in minced pork can be explained by a high content of anthocyanins and phenolic acids. Anthocyanins cause the red, purple, blue, and black colors of various plant materials and are the major class of phenolics in blackcurrants [34]. There is evidence that BC and some other high-value berry pomaces are rich in polyphenolic antioxidants, and when included in the composition of meat products were able to slow down oxidation and inhibit the growth of both pathogenic and spoilage bacteria [13,14,21]. The high AO activity of BC, followed by CB and RB powders, was also confirmed in the present study. Furthermore, the results showed that anthocyanin-rich plant materials, such as BC, CB, and RB, are more efficient free radical scavengers than those containing fewer or no anthocyanins. It can be explained by the fact that this activity of phenolic compounds is strongly dependent on their molecule structure, mainly the number and localization of hydroxyl groups attached to an aromatic ring.

In the present study, a hypothesis can be made based on the data in Table 2 that for six plant powders, free radical scavenging forms the backbone of the antioxidant effect of the polyphenols. This conclusion can be made from the strong positive correlation between AO and TPC (r = 0.98) if chokeberry CB and tomato TO are excluded from the calculation; when they are included, the r value drops to 0.68, indicating that with CB and TO, polyphenols may have relatively lower importance in radical scavenging in their overall antioxidant effect. That hypothesis can be tested in the next step of our studies, using different methods for measuring AO capacities. Even though Badarinath et al. [35] and Burri et al. [36] have seen very similar results when comparing different methods for AO measurements, it may depend on the selection of plants in the study. In our case, the different mechanisms of each plant’s AO capacities have to be looked into in future studies. According to Skrovankova et al. [37] the overall antioxidant capacity may be clarified by insight into the connection of different bioactive compounds (organic acids, vitamins, polyphenols, and carotenoids), working additively or synergistically through different mechanisms in relation to the total antioxidant capacity The low radical scavenging ability of bioactive compounds of GAR or RHB causes relatively high counts of radicals to be continuously produced by oxidative reactions in the meat matrix. These radicals can inhibit microbial growth by creating oxidative stress that causes damage to a variety of microbial macromolecules, including antioxidant enzymes superoxide dismutase (SOD), catalase and glutathione peroxidase, and DNA, and ultimately lead to microbial cell death [38]. In addition, other antibacterial effect mechanisms of additives are combined with the free radical scavenging effect of polyphenols and other scavengers.

Recent model membrane experiments by Wyżga et al. [39] showed that BC extracts affected the bacterial pathogens model systems by penetrating the lipid systems and decreasing their condensation and altering their morphology. Zhao et al. [40] revealed that the antibacterial effect against *Listeria monocytogenes* was associated with an aglyconic dihydrochalcone phloretin, which decreases the intracellular protein content due to DNA aggregation. In our study, phloretin-di-C-hexoside was found in BC-, CB-, and TO-supplemented minced pork and beef, and this compound, as shown by Barreca et al. [41], may also have had an inhibitory effect on tested microbial counts in minced pork with BC powder.

The effect of the other plant powders on the average total count of tested microorganisms was not significantly different from the controls. In addition, the supplemented minced pork and beef samples had quite similar polyphenolic profiles and similar percentage distributions of the polyphenolic compound classes, such as anthocyanins, dihydrochalcones, flavanols, flavonols, hydroxycinnamic acids, and organic acids, on days 1 and 8.

However, higher microbial counts were observed in RB samples during storage, and this could be explained by the higher pH (5.8) and water activity (0.988) of RB-supplemented minced meat samples compared to the other samples studied. Contrary to our study, Bobinaité et al.’s [42] study showed that the extracts of RB pomaces inhibited the growth of Gram-positive bacteria due to the high diversity of the polyphenolic compounds in the tested extracts. Although the content of phenolic compounds in the rowan berries depends on the cultivated species, the major phenolic classes in these berries are anthocyanins and flavonols [12]. CB is a rich source of anthocyanins and hydroxycinnamic acids, especially chlorogenic acids, which also have antibacterial properties [43]. These compounds were also detected in the present study, but the antimicrobial effect on the tested microorganisms was weak. TO extracts have been shown to have an antimicrobial effect against *P. aeruginosa* [44], although this was not confirmed for the tested microorganisms in the present study. Szabo et al. [44] found that the extracts of the TO processing byproducts contain, in addition to other polyphenols, isochlorogenic (3,5-dicaffeoylquinic) acid, which correlated with the antibacterial activity against Gram-positive bacteria. However, the antibacterial properties of polyphenols may depend on interactions between the polyphenols and the bacterial cell surface [45]. Mohammed and Mustafa [46] identified four new furanocoumarins from apple seeds, which have promising antibacterial activity against *P. aeruginosa* and other bacteria. Kapp et al. [10] found that the main polyphenols in apples were quercetin galactoside, procyanidin B1, and (epi)catechin trimer in peels, chlorogenic acid in flesh, and phloridzin in seeds. Similarly, the present study found quercetin pentosides and rhamnosides, procyanidin B1, and chlorogenic acids in APL and APLS samples. Both APL and APLS powders had similar polyphenolic profiles, but did not have a statistically significant antimicrobial effect against the total microbial counts in our study. Furthermore, the total microbial counts in the minced meat samples supplemented with APL and APLS slightly increased compared to the controls (Figure 4a,b), except for the slight inhibitory effect of APL on the counts of yeasts and molds in minced beef on days 6 and 8, compared to APLS or control samples (Figure 3B and Figure 4f).

Generally, the low microbial growth inhibition effect of the above-mentioned powders can be explained by the addition of only 2% of plant powder to the minced meat samples in this study. The amount of powder was likely too low to achieve a significant effect. This is also confirmed by our previous study [18], where the minimal inhibition concentrations of the extracts of CB, BC, and RB berries and their pomaces against the tested pathogens were high.

In addition, the microbial growth inhibition effects of plant powders in minced pork and beef may also depend on the interaction between polyphenols and other food constituents. Weiss et al. [47] concluded that the decrease in antibacterial activity in the food matrix is mainly a physical phenomenon and can be explained by the many molecular interactions between the compounds added to a structured food and food composed of different ingredients. Bouarab-Chibane et al. [48] found that due to the heterogeneous structure of perishable foods, the antibacterial molecules are heterogeneously distributed, and the exposure of the polyphenolic components to fat can reduce its antibacterial activity in the food matrix.

The food formats derived from fruit and berry production can be juice, powder, extract, and pomace. All these may have prebiotic and pharmacological properties, and therefore, have good potential for industrial-scale production. Reprocessing byproducts from juice production e.g., pomaces for highly nutritional and health-beneficial substances, is in full agreement with zero waste conception and the circular bioeconomy, which is currently an important worldwide trend.

## 5. Conclusions

Similar microbial growth dynamics, polyphenolic profiles, and percentage distributions of the polyphenolic compound classes were observed in minced pork and beef samples supplemented with plant powders within the study period. RHB powder had the strongest microbial growth inhibition effect in both minced meats, followed by BC pomace powder in minced pork. The findings showed that the addition of RHB powder to minced meat products reduced the average total microbial counts by 10-fold, and the counts of *Pseudomonas* spp., yeasts, and molds by 100-fold over the entire shelf life compared to the control sample. Also, the total microbial counts in minced meat samples supplemented with other plant powders were generally lower than in the control. The highest antioxidative capacity was found in anthocyanin-rich BC and CB pomace powders. The study results proved that all investigated plant powders used can be applied for the valorization of minced meat products. Different plant materials can exhibit both antimicrobial and antioxidant effects.

## Figures and Tables

**Table 1:** Compounds tentatively identified by LC-MS in the plant powders, minced pork, and beef samples supplemented with plant powders on days 1 and 8 of the experiment.

Pseudomolecular Ion Mass-to-Charge Ratio (*m*/*z*)	Compound	Detected in Tested Samples *						
		BC	CB	RB	APL	APLS	TO	RHB
**Anthocyanins:**								
417.0827; 435.0933	Cyanidin pentosides		+●○▲∆	+●○▲∆				
447.0928; 465.1038	Cyanidin hexoside	+●○ ▲∆	+●○▲∆	+●○▲∆				+ ▲∆
593.1506; 611.1618	Cyanidin rutinoside	+●○ ▲∆					+	+
463.0882; 481.0988	Delphinidin glucoside	+●○ ▲∆						
609.1461; 627.1567	Delphinidin rutinoside	+●○ ▲∆						
**Dihydrochalcones:**								
435.1297	Phloridzin	+●○	+●○	+●○	+●○▲∆	+●○ ▲∆	+	+●○
597.1825	Phloretin-di-C-hexoside	+●○ ▲∆	+●○▲∆	+●○▲∆	+	+	+●○▲∆	
**Flavanols:**								
289.0718	Catechins	+●○ ▲∆	+●○▲∆	+●○▲∆	+●○▲∆	+●○ ▲∆		+●○▲∆
305.0700	(epi)gallocatechin	+						
577.1352	Procyanidin B-type	+	+	+	+●○▲∆	+●○ ▲∆		+
865.1985	Procyanidin C-type		+	+	+	+		+
**Flavonols:**								
301.0354	Quercetin	+●○ ▲∆	+●○▲∆	+●○▲∆	+	+	+●○ ∆	+● ▲∆
433.0776	Quercetin pentosides	+●○	+●○▲∆	+●○	+●○▲∆	+●○ ▲∆		+●○
447.0928	Quercetin rhamnoside	+●○ ▲∆	+●○▲∆	+●○▲∆	+●○▲∆	+●○ ▲∆	+	+●○▲∆
463.0882	Quercetin hexosides	+	+●○▲∆	+●○▲∆	+	+	+	+ ▲∆
463.0882	Aromadendrin glucuronide		+	+				+
595.1305	Quercetin pentosyl-hexosides		+●○▲∆	+●○▲∆			+●○	
593.1506	Kaempferol rutinoside	+	+	+			+	+
609.1461	Quercetin rhamnosyl hexoside	+●○ ▲∆	+●○ ▲∆	+●○ ▲∆	+●○	+●○	+●○	+●○
625.1410	Quercetin dihexosides	+	+●○▲∆	+●○▲∆				+
741.1884	Quercetin pentosyl rutinoside			▲∆			+● ▲∆	
771.1989	Quercetin hexosyl rutinoside						+● ▲∆	
**Hydroxycinnamic acids:**								
153.0193	Protocatechuic acid	+●○ ▲∆	+●○▲∆	+●○▲∆	+○ ▲∆	+○ ▲∆	+●○▲∆	+
337.0929	Coumaroylquinic acids	+●○ ▲∆	+●○▲∆	+●○▲∆	+●○▲∆	+●○ ▲∆	+ ▲∆	
341.0878	Caffeic acid glucosides	+● ▲∆			+	+	+● ▲∆	+
353.0873	Chlorogenic acids	+●○ ▲∆	+●○▲∆	+●○▲∆	+●○▲∆	+●○ ▲∆	+●○▲∆	
515.1195	Di-caffeoylquinic acids		+●○▲∆	+●○▲∆	+●○▲∆	+●○ ▲∆	+●○▲∆	
**Organic acids:**								
147.0299	Citramalic acid				+●○ ▲∆	+●○ ▲∆		
191.0197	Citric acid	+●○ ▲∆	+●○▲∆	+●○▲∆	+●○▲∆	+●○ ▲∆	+●○▲∆	+●○▲∆
191.0556	Quinic acid	+●○ ▲∆	+●○▲∆	+●○▲∆	+●○▲∆	+●○ ▲∆	+●○▲∆	+

* Plant powders (+), minced pork on day 1 (●) and day 8 (○), and beef samples on day 1 (▲) and day 8 (∆) supplemented with different powders: blackcurrant (BC), chokeberry (CB), rowan berries (RB), apple, seeds removed (APL), apple with seeds (APLS), tomato (TO), garlic (GAR), and rhubarb (RHB).

**Table 2:** In vitro antioxidative (AO) capacity of plant powders in descending order of free radical DPPH scavenging assay, expressed in Trolox equivalents (TE) in mM per gram of dry weight (DW). Total phenolic content (TPC) is in mg gallic acid equivalents (GAE) per g of DW.

Plant Material	Abbreviation	AO mM TE/DWg	TPC mg GAE/DWg
Blackcurrant	BC	130 ± 8.7	24.6 ± 0.07
Chokeberry	CB	127 ± 25.9	55.0 ± 1.29
Rowan berries	RB	119 ± 0.6	19.9 ± 0.06
Apple with seeds	APLS	105 ± 4.4	13.3 ± 0.04
Apple, seeds removed	APL	104 ± 4.1	12.7 ± 0.04
Rhubarb	RHB	88 ± 9.2	10.5 ± 0.05
Tomato	TO	75 ± 3.2	16.1 ± 0.05
Garlic	GAR	72 ± 5.5	3.9 ± 0.01

There was a strong positive correlation (r = 0.98; *p* < 0.001) between AO and TPC if chokeberry and tomato were excluded from the calculation, otherwise r = 0.68 (*p* < 0.05) including all supplements.

## Data Availability

The original contributions presented in the study are included in the article, further inquiries can be directed to the corresponding author.
